# Low back pain around retirement age and physical occupational exposure during working life

**DOI:** 10.1186/1471-2458-11-268

**Published:** 2011-04-28

**Authors:** Sandrine Plouvier, Julie Gourmelen, Jean-François Chastang, Jean-Louis Lanoë, Annette Leclerc

**Affiliations:** 1 Inserm u1018, Epidemiology of occupational and social determinants of health, CESP, Inserm, Villejuif, France, University of Versailles st-Quentin, URMS 1018, Versailles, France

## Abstract

**Background:**

Physical occupational exposure is a risk factor for low back pain in workers but the long term effects of exposure remain unclear. As several countries consider increasing the retirement age, further information on this topic is relevant. This study aimed to describe the prevalence of low back pain among middle aged and aging individuals in the general French population according to physical occupational exposure and retirement status.

**Methods:**

The study population originated from the French national survey 'Enquête décennale santé 2002'. Low back pain for more than 30 days within the previous twelve months (LBP) was assessed using a French version of the Nordic questionnaire. Occupational exposure was self assessed. Subjects were classified as "exposed" if they were currently or had previously been exposed to handling of heavy loads and/or to tiring postures. The weighted prevalence of LBP was computed separately for men and women, for active (aged 45-59) and retiree (aged 55-74), according to 5-year age group and past/present occupational exposure.

**Results:**

For active men, the prevalence of LBP was significantly higher in those currently or previously exposed (n = 1051) compared with those never exposed (n = 1183), respectively over 20% versus less than 11%. Among retired men, the prevalence of LBP tended towards equivalence with increasing age among those previously exposed (n = 748) and those unexposed (n = 599).

Patterns were quite similar for women with a higher prevalence in exposed active women (n = 741) compared to unexposed (n = 1260): around 25% versus 15%. Similarly, differences between previously exposed (n = 430) and unexposed (n = 489) retired women tended to reduce with age.

**Conclusion:**

The prevalence of LBP in active workers was associated with occupational exposure. The link with past exposure among retirees decreased with age. These results should be considered for policies dealing with prevention at the workplace and retirement.

## Background

In most developed countries, the population is expected to age in the next decades. For instance, it is expected that one third of the French population will be older than 60 in 2060 [1]. The growing aging population leads governments to rethink employment and retirement policies. Staying in the labor force despite approaching retirement age, and increasing the retirement age, are two common patterns being explored [2]. Taking into account past physical exposure for defining age at retirement is also considered in several countries.

It has been suggested that older people experienced a higher prevalence of episodes of severe back pain [3] and that persistence of low back problems was more frequent with increasing age [4]. Physical occupational exposure is considered as a risk factor for low back pain among the working population, even if a debate still exists about the level of evidence [5-7]. However, the long term effects of exposure to occupational risk factors, and its delayed effects once exposure has ceased are not well known. Nonetheless, some studies - based on small samples - do suggest that retired individuals could suffer from low back pain related to previous occupational strain several years after exposure had ceased [8-11]. This topic is difficult to investigate in countries where workers suffering from chronic low back pain are able to receive a disability pension or retire because of disability. In France, the situation is different, since disability retirement does not exist [12].

Our objective was to study the prevalence of low back pain among middle aged and aging individuals from a large sample issued from the general French population according to past or present physical occupational exposure and retirement status (active or retired).

## Methods

### Population

#### The 'Enquête décennale santé 2002'

Every ten years, a national population-based survey on health is conducted in France. The 'Enquête décennale santé 2002' (EDS 2002), took place between October 2002 and September 2003 [13] with the aim to evaluate care seeking, prevention behavior, and several other dimensions of health. For its purpose, a random sample of the French population was obtained from the files of INSEE, the National Institute for Statistical and Economic Studies, in charge of censuses and compulsory surveys. Each study participant was interviewed on three separate occasions at home by a trained interviewer. At the first interview, participants aged at least 18 and considered able to complete a questionnaire were asked to complete a self-administered questionnaire to be handed back at one of the subsequent visits. This questionnaire included questions on health, health behaviors, and occupation (physical exposures, psychosocial strains, work organization).

The survey was performed with the approval of the appropriate committees for this kind of survey in France: CNIS (Conseil National de l'Information Statistique) and CNIL (Commission Nationale de l'Informatique et des Libertés).

Those living in a nursing home or a retirement home were not contacted, and those considered unable to complete a questionnaire (about 1% of the target population) were excluded from the corresponding part of the survey.

### The study population

The study population included the participants to the EDS 2002 aged between 45 and 74 years, who were either employed or retired at the time of the survey, had participated in all three interviews and answered the questions on low back pain and the two questions on occupational exposure in the self-administered questionnaire. Individuals in employment who had not been working for health reasons for a period of several weeks at the time of the survey were excluded, as were those who had retired before the age of 55. We also excluded those aged 60 years or older who were still employed since age at retirement is rather low in France and those who are still active beyond 60 years represent a selected population.

### Measurements

#### Low back pain

A French version of the Nordic questionnaire for the analysis of musculoskeletal symptoms [14] was used to assess low back pain. In the self-administered questionnaire, the lower back was pictured and low back pain was defined as ''pain, discomfort or disability in the lower back", the region indicated on the picture, "whether or not the pain radiates to the leg''. Subjects were asked if they had experienced low back pain in the preceding twelve months. If the answer was "yes", they had to indicate the cumulative duration of low back pain during the past 12 months: 1-7 days, 8-30 days, 30 days but not every day, or every day. We grouped the last two categories in order to study low back pain which lasted for more than thirty days during the past twelve months (LBP), which can be considered as frequent or recurrent pain [15].

### Occupational exposure

The self-administered questionnaire included two questions on physical exposure at work: whether the work involved carrying heavy loads, and whether it involved tiring postures. For each of these two occupational strains, active workers indicated if they were currently exposed, if they had been exposed in the past but were no longer exposed, or if they had never been exposed. For retired subjects, the exposure, if any, was exposure during their active working life. A subject was classified as "exposed" to physical occupational strains if he or she had been exposed in the past, or was exposed at the time of the survey, to at least one of the two occupational strains.

### Retirement status

Retirement status was assessed during the face-to-face interview.

### Analyses

The prevalence of low back pain for more than 30 days in the previous year was computed separately for men and women, for active and retired, according to 5-year age group and past/present occupational exposure. In this national study, weightings taking into account the study design were available. Both weighted and unweighted prevalences were calculated. Results presented here are weighted prevalences and their 95% confidence intervals, giving estimates for the whole population. Prevalences for ever exposed and never exposed subjects were compared within sex, age and retirement status subgroups. Comparison tests were based on the distribution of the estimates, with an assumption of normality for the weighted prevalences.

## Results

### The study population included 3581 men and 2920 women (Tables 1 and 2)

**Table 1:** Working status and occupational exposure according to age among men

Age	45-49	50-54	55-59	60-64	65-69	70-74
Exposed active	418	414	219			
Unexposed active	463	465	255			
Exposed retired			55	232	262	199
Unexposed retired			60	201	188	150

**Table 2:** Working status and occupational exposure according to age among women

Age	45-49	50-54	55-59	60-64	65-69	70-74
Exposed active	310	286	145			
Unexposed active	558	458	244			
Exposed retired			32	126	142	130
Unexposed retired			63	153	134	139

**Figure 1:**
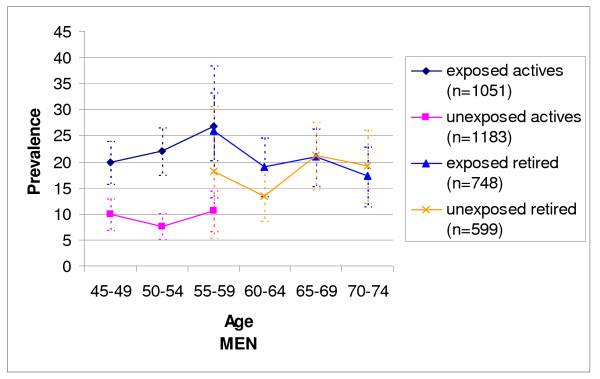
**Prevalence of Low back pain for more than 30 days within the previous 12 months and its 95% Confidence Interval among men according to age and work status**.

Concerning men (Figure 1) below the retirement age (aged 45-59), the prevalence of LBP was significantly higher in all three age groups among those currently or previously exposed to manual material handling and/or tiring postures (n = 1051) compared with those never exposed to these strains (n = 1183), respectively over 20% versus less than 11% (p < 0.0001 for each of the three age groups).

Among retired men (aged 55-74), the differences in LBP prevalence between those previously exposed (n = 748) and those never exposed in the past (n = 599) tended to become smaller with increasing age. No difference was observed for those aged 65 and over and, for the oldest age group (70-74 years) the prevalence was even slightly higher among unexposed men.

**Figure 2:**
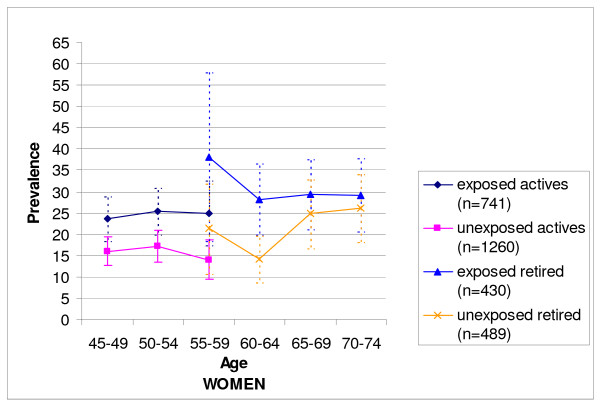
**Low back pain for more than 30 days within the previous 12 months and its 95% Confidence Interval among women according to age and work status**.

The patterns were quite similar for women (Figure 2). Among active women exposed to physical strains at work (n = 741), the prevalence of LBP was around 25% compared to around 15% for those never exposed (n = 1260). These differences, although less pronounced than those observed for men, were also significant in the three age groups (p < 0.02 in the three age groups). For retired women, the differences between those exposed in the past (n = 430) and those never exposed (n = 489) also tended to decrease with age, as indicated in Figure 2. In the 60-64 age group, the prevalence among exposed retired women was nonetheless significantly higher than that for their never exposed counterparts (p < 0.05).

Among the 55-59 years age group comprising both active and retired subjects, those retired more often declared that they suffered from LBP than their active counterparts, except for men classified as 'exposed' who had a similar prevalence whether they were retired or not.

## Discussion

In this population, the role of occupational exposure appeared to be important during a person's working life. Among retired individuals however, the link between past exposure and LBP tended to become weaker with increasing age. Nonetheless, differences in the prevalence of LBP between exposed and never exposed in the past still existed for young retirees.

### Before discussing these results, we will consider methodological issues of this study

Since this study is cross sectional, the temporal sequence between LBP and exposures is unknown and the possibility of bias must be considered. Individuals who suffered from LBP could have moved to less physically strenuous jobs prior to the survey. This selection effect would have lead to an overestimation of LBP in the 'unexposed' group if only occupational exposure at the time of the survey had been considered. To minimize this bias, active subjects were classified as "exposed" to occupational strains not only if they were exposed at the time of the survey, but also if they had been exposed in the past. It might be that some subjects had never been exposed because they suffered from LBP very early in their life, before the beginning of their work history, but this must be infrequent. A recall bias could however have occurred with those suffering from LBP having overestimated their exposure to occupational risk factors. Differential errors are expected to be limited since the questionnaire covered many areas of health. In addition, in France there is no specific category such as work-related low back pain, except in very special situations. Among older people, non differential errors could also occur.

Exposure to the occupational strains studied here occurs more often in the lowest occupational classes [16] known to have a shorter life expectancy [17]. Older retired individuals who had been exposed in the past may therefore have been underrepresented in the present study. However since common LBP is associated with disability rather than with mortality this should not be an important source of bias. In addition, we did not consider people living in nursing home or retirement home but few people at these ages currently live in these situations in France [18]. Active workers above 60 years of age were also not studied in order to minimize selection bias.

Exposures were self assessed which is the only option available when the entire occupational history is considered, especially in the general population. The questions used may be considered as not very specific. The level of exposure is not precisely known. However, rather simple questions about various aspects of the demand of physical work perform rather well as to reproducibility and validity in workers [19]. We are not aware of comparable data on the assessment of the past occupational exposure of retirees. For working people the frequency of exposure to handling of heavy loads, was consistent with that found in a French national survey on working conditions which took place in 2003 [20], although a precise comparison is difficult due to the age structure in our population. As far as we are aware, there is no such information for retirees within the French population.

Among men, missing data for occupational exposure were more frequent for farmers (compared with blue collar workers) and less common among those in management and intermediate occupations. Being older increased the probability of non response to these questions among active subjects; among retired individuals, the only group with a significantly increased frequency of missing information was the oldest. Among women, farmers also answered less often compared to women in other occupations and non response was significantly more frequent only among the oldest active women and increased with age among those retired. There was no difference in LBP status between those who answered these questions and those who did not in either group.

We wanted here to present descriptive data raising questions on the long term effects of occupational exposure on LBP rather than quantify these effects. For that reason, potential confounding factors, such as obesity, were not taken into consideration. Even though the prevalence of such factors could differ between subgroups, adjustments are not expected to modify the main results. Non-occupational physical activities were not considered. There is little information about physical activity after retirement, especially in relation with physical activity at work [21-23]. In one study, the largest gain in sport score was observed among those who were the least active at work, however it was not possible to conclude to gain or loss in physical activity in general [21]. Does the activity performed by retired individuals differ according to the physical demand they experienced at work? And if it is the case, how does it differ? To our knowledge this topic has not been investigated.

Finally, with data from a cross sectional survey, comparisons between age groups might be due to a generation effect. However, this could not explain the main results which were based on comparisons between exposed and unexposed individuals within the same age group.

Our results do tend to indicate that early prevention in occupational field is of importance not only for short term effects, but also for long term effects when workers retire.

In the 55-59 years age group comprising both active and retired individuals, the figures for LBP prevalence are consistent with health playing a role in the decision to retire [24]. However, the exposed men in this age group have a high prevalence irrespective of their situation, active or retired.

Previous studies have reported the long term effects of occupational exposure on low back pain. In Sweden low back pain was increased among Post Office pensioners, aged 71 to 75 years, who had been exposed to the manual handling of heavy loads for over twenty years [10]. Several types of low back pain have been related to previous biomechanical strains within a sample of the Gazel cohort comprising both older active workers and 'young' pensioners [25]. Manual shipyard workers were also found to suffer from musculoskeletal disorders two to three years after retirement, attributable to heavy physical workload during their active life [8]. In the latest study, retired office workers were found to suffer from slightly more back symptoms than three years earlier. Locomotor impairment of the lower back was also associated with the duration of work at the coal face in miners retired for at least 10 years [11]. In another study carried out in France with a 5 year follow-up of retirees from various occupational settings, the lifetime physical workload was associated with frequency and course of musculoskeletal pain at various sites of the body [9]. A significant increase in pain prevalence after five years for some of the 'unexposed' subjects was also observed.

Many previous studies were based on small samples [8,9,11]. Other ones considered only young retirees [8], or included active workers [25]. In the one dealing with low back pain among older retired subjects, the long term effects were observed only for those with the longest duration of exposure [10]. The other studies focused on an outcome somewhat different including locomotion impairment [11] and pain at various sites [9]. Our results are globally in accordance with these previous studies, with the advantage of being based on a large population sample.

In France, the legal age of retirement, which was 60 when the survey was performed, is rather low compared with other western countries. Workers close to retirement age were also often out of the labour market due to employment policies or for economic reasons. Hence, generalization of findings from France to other countries might be discussed. However, the fact that the effects of past physical occupational exposures do not disappear with retirement is probably a general result which would be observed also with an older age at retirement. Furthermore, being exposed at older age could also have specific consequences on the lower back. Considering that retiring later implies a longer duration of exposure, at least for a part of the workers, these aspects appear important to consider, especially as public policies are favouring increasing age at retirement.

## Conclusion

Exposure to occupational strain plays an important role for recurrent or persistent low back pain among active workers. Among the exposed and unexposed retirees, the prevalence of LBP tended towards equivalence with increasing age in this national survey. Further studies with a longitudinal design are needed to confirm this observation, to explore the underlying mechanism and to quantify more precisely the delayed effects of exposure.

## Competing interests

The authors declare that they have no competing interests.

## Authors' contributions

SP performed the analyses and wrote the manuscript. JG was involved in the analyses and the revision of the manuscript. JFC revised the manuscript. JLL was involved in the conception and design of the EDS. AL was involved in the acquisition of data, the design of this study and the revision of the manuscript. All authors read and approved the final manuscript.

## Pre-publication history

The pre-publication history for this paper can be accessed here:

http://www.biomedcentral.com/1471-2458/11/268/prepub
